# The time is ripe for oocyte in vitro maturation

**DOI:** 10.1007/s10815-021-02209-x

**Published:** 2021-05-08

**Authors:** Carlos E. Plancha, Patrícia Rodrigues, Mónica Marques, Joana M. Almeida, Paulo Navarro-Costa

**Affiliations:** 1 Instituto de Histologia e Biologia do Desenvolvimento, Faculdade de Medicina, Universidade de Lisboa, Lisboa, Portugal; 2 Centro Médico de Assistência à Reprodução (CEMEARE), Lisboa, Portugal; 3 Escola de Psicologia e Ciências da Vida, Universidade Lusófona de Humanidade e Tecnologia, Lisboa, Portugal; 4 Instituto Gulbenkian de Ciência, Oeiras, Portugal; 5 Instituto de Saúde Ambiental, Faculdade de Medicina, Universidade de Lisboa, Lisboa, Portugal

**Keywords:** IVM, Oncofertility, Fertility preservation, ART, Oocyte, Oocyte maturation

When it comes to welcome transitions, 2021 seems to have started on a promising note. From the ushering in of COVID-19 vaccination programs to a renewed appreciation of evidence-based policy in many countries, progress manifested itself in swift and emphatic manners. The same can be said of the field of assisted reproduction techniques (ART), home to a recent practice committee document on oocyte in vitro maturation (IVM) [1]. In this landmark document, the practice committees of the American Society for Reproductive Medicine (ASRM), the Society of Reproductive Biologists and Technologists, and the Society for Assisted Reproductive Technology (SART) present a considerable (and compelling) body of published evidence supporting the conclusion that IVM should no longer be considered an experimental technique.

This coming of age for IVM may be a surprise to many. Indeed, the initial attempts of promoting meiotic resumption under fully controlled conditions were met with fairly underwhelming results. On hindsight, we can speculate whether our knowledge of oocyte physiology and development was, at the time, sufficiently mature to support the design of truly effective IVM. Quite tellingly, it was precisely an investment on the basic aspects of reproductive biology — particularly on how intrafollicular cell signaling primes the oocyte to successfully navigate past its prophase I arrest — that opened the door to a new generation of more efficient IVM protocols [2–5]. Regardless of the hows and whys, it is clear that IVM did not translate, from the start, into an immediate clinical success like ICSI, and that initial wave of disappointment has clouded the technique ever since.

**Fig. 1:**
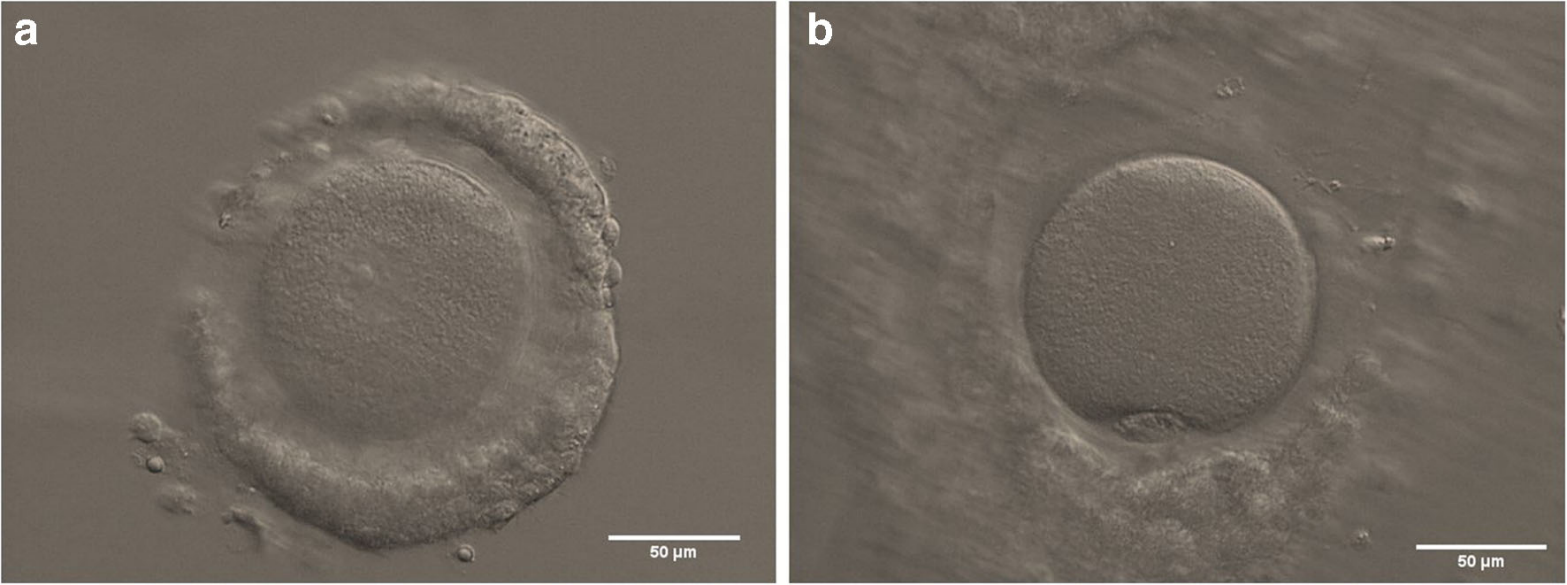
**a** Non-expanded human cumulus-oocyte complex (COC) containing an immature oocyte, as indicated by the presence of a clearly visible germinal vesicle; **b** in vitro maturation-derived human metaphase II oocyte with an extruded first polar body, enclosed in an expanded COC. Scale bars: 50 μm

In light of the above, the transition of IVM from a niche approach to mainstream ART has not been a smooth ride. Other, more practical, reasons have also complicated this transition. For starters, the technical aspects, of the procedure: the small size of the targeted antral follicle population makes oocyte retrieval more challenging. Likewise, isolating non-expanded immature cumulus-oocyte complexes (COCs; Fig. 1) from an often convoluted mix of cellular debris and body fluids demands greater training and technical proficiency from the clinical embryologist. This additional workload for both medical doctors and clinical embryologists may have hindered a faster adoption of IVM across the board, particularly in light of the lower numbers of transferable embryos associated with the technique (a reflection of the increased attrition to get from immature COCs to high-quality embryos). Such technical aspects have been further compounded by an inconsistent (and sometimes outright anarchic) use of the term IVM, an acronym that has been employed to label procedures ranging from mild ovarian stimulation to the extended culture of priming-irresponsive immature oocytes denuded of their cumulus cells [6]. This inconsistent terminology complicates inter-study comparisons and obfuscates the difference between valuable procedures and those that should be considered largely inefficient at best.

Again, it is important to emphasize that IVM protocols have been significantly improved in the last few years, translating into higher take-home baby rates and a more informed appreciation of their overall safety [7, 8]. Among these recent advances, the inclusion of a pre-maturation step (pre-IVM) with c-type natriuretic peptide stands out as particularly noteworthy [9]. The basis of this biphasic approach to IVM is to improve oocyte competence by blocking the premature spontaneous meiotic resumption typically associated with the release of COCs from the antral follicle microenvironment. Indeed, by temporarily maintaining the prophase I meiotic arrest until oocytes are transferred to the IVM medium, the pre-IVM step promotes a more harmonious coordination between the nuclear and cytoplasmic maturation steps required for the formation of a developmentally-competent female gamete. In addition, the pre-IVM incubation period opens the door to the use of supplements that may prime COCs to respond more effectively to the subsequent stimuli induced by the IVM medium.

Ultimately, which patients will benefit from a more widespread use of IVM? Two obvious groups emerge: (i) women prone to developing adverse reactions to standard follicular priming and (ii) women that cannot wait for the entire duration of a priming regimen due to the urgency of initiating gonadotoxic cancer treatment. Accordingly, IVM has proven itself a valuable technique both to prevent ovarian hyperstimulation syndrome and as a safeguard of the reproductive potential of oncofertility patients [10, 11]. Moreover, the possibility of repurposing some of the basic principles of IVM to minimal priming regimens may increase the number of available oocytes per cycle while ensuring a more physiologically balanced ovarian stimulation. In particular, IVM can be used to promote meiotic resumption in timely retrieved immature COCs after low-dose FSH stimulation. Such approach would overcome the main limitation of minimal priming regimens (lower number of mature gametes obtained per cycle), thus potentially opening IVM to a wider population of patients.

Like any other ART procedure, IVM will face, as it moves forward, three outstanding issues: (i) to become easily available to those in need; (ii) to improve its effectiveness based on robust clinical trials; and (iii) to monitor the safety and well-being of patients and offspring. Indeed, as access to IVM becomes more commonplace, so does the need for thorough longitudinal studies, particularly those directed at evaluating the development of children conceived after this technique. In this regard, the use of national registries has been an important aspect in the steady increase of follow-up studies of children born from different ART procedures. Of note, such studies have been largely encouraging for the use of another technique — oocyte cryopreservation — that not so long ago was also considered experimental [12, 13].

As before, basic science will be a crucial ally in these battles: effectiveness and safety will surely benefit from a deeper understanding of how the oocyte’s molecular machinery promotes meiotic progression while simultaneously preparing for the maternal control of early embryogenesis. In this regard, evolution has been generous to the IVM cause: like in humans, oocytes from species as diverse as worms, insects, and rodents all temporally arrest their development at prophase I [14]. The deep evolutionary conservation of the discontinuous female meiotic program (a likely requirement for the complex coordination between oocyte growth, meiosis, and fertilization) means that the use of model organisms can greatly accelerate the discovery of the fundamentals of oocyte maturation. Indeed, core meiotic regulators such as the CDK1/Cyclin B complex, cyclic AMP, and ERK signaling have been shown to exert similar functions in oocytes from very diverse species [15]. Recent developments on how the epigenetic regulation of meiotic chromosomes can impact oocyte quality have also opened up exciting new avenues of research [16].

In conclusion, we believe that this joint practice committee document on IVM is of the upmost importance for the field of Reproduction Medicine. A more widespread use of IVM will be fundamental to meet the increasing demand for oncofertility preservation, while offering alternatives for a more sustainable and physiological use of hormonal stimulation. Accordingly, we expect that this document will rekindle clinical interest in IVM and motivate funding bodies to further invest in refinements to the procedure. If ART clinics are aware of the specific technical requirements underlying an effective IVM program and recognize the procedure’s essential role in safeguarding the reproductive potential of a growing number of couples, the result will surely be to the benefit of all stakeholders.
